# Ethical collection of animal cadavers for veterinary education

**DOI:** 10.1186/s12917-025-04543-z

**Published:** 2025-02-24

**Authors:** Edwin Louis-Maerten, David M. Shaw

**Affiliations:** 1 Institute for Biomedical Ethics, University of Basel, Basel, Switzerland; 2 Care and Public Health Research Institute, Maastricht University, Maastricht, The Netherlands

**Keywords:** Body donation, Domestic animals, Veterinary education, Donor program, Ethics

## Abstract

The use of animal cadavers in initial and continuing veterinary education is widely accepted, but ethical considerations regarding how to obtain and use them are often lacking. It can be argued that the use of animal cadavers should be guided by principles similar to those regulating the collection of human cadavers for scientific purposes. In humans, the use of unclaimed cadavers from unknown provenance, cadavers from criminals or homeless people, or cadavers robbed from their grave no longer happens in most countries. Accordingly, body donation programs have been emphasized to be the sole acceptable source of cadavers for medical education. The purpose of this article is to argue that this should also be the case for domestic animals, contrary to the current practices of using unwanted animals or animals bred for this purpose. But having a body donation program is not enough to make it ethical: care should also be taken in order to include principles such as informed consent from the owners, the absence of commercial uses of the bodily remains and the respect for all parties involved in the process. Overall, the importance of maintaining respect towards the reception and use of a donated cadaver in all circumstances should be the ethical priority for students and practitioners. By applying such principles, we can better ensure that the use of animal cadavers in education and training is transparent, respectful and responsible.

## Background

In human and veterinary medicine, initial and continuing training sometimes requires direct contact with living bodies, cadavers or tissues. Some argue for example that dissection courses are a core tool for teaching human anatomy and surgical techniques, especially because it conveys macroscopic knowledge in a didactic fashion and because it stimulates the learning and practicing of manual skills [1–2]. In veterinary medical curricula, dissection is also considered as an “excellent tool for clinical skills training and for the practice of surgical techniques, once basic competency has been gained using non-animal alternatives” [3]. In theory, dissection courses could be completely replaced by alternative methods such as mannequins or computer models, but there is still a perception that the use of cadavers is necessary in the learning process of veterinary students [2, 4–6]. For the sake of the argument, let us consider the use of animal cadavers as indeed being favorable for training purposes in veterinary sciences. By veterinary education, we will include formal training during initial and continuing education, but also informal individual or collective self-study (see below for a case study).

The question is: what would be best practice in terms of obtaining such cadavers? Contrary to the collection of human cadavers for medical science and training, there are few to no laws regulating this practice in veterinary medicine, and very little ethical discussion on the topic. The aim of this article is to bridge this gap by exploring the requirements for an ethical sourcing of cadavers for veterinary education. In a first part, we will discuss the moral grounds of the current practice for cadaver collection in veterinary education, and why body donation programs should be the preferred option. In a second part, based upon a case study, we will explore additional considerations for ethical donation of animal cadavers. In a third part, we will review the ethical requirements needed to obtain a human cadaver for medical education to better inform the case of domestic animals. Finally, we will discuss how these requirements can be applied to veterinary education, and suggest some guidance for ethical cadaver collection in domestic animals.

## What is an ethical source of animal cadavers for veterinary education?

In veterinary training programs, three different sources of cadavers may be distinguished: (1) cadavers from usually healthy but unwanted animals (e.g. from animal shelters), (2) cadavers from purpose-bred individuals, and (3) cadavers donated by owners after natural death, accident or euthanasia [7–8]. But these three different sources are not ethically equivalent, and we will argue that ethical concerns may be raised regarding the first two sources. This means that, as for humans [9–11], body donation programs should become the sole acceptable source of cadavers for veterinary education.

If we first focus our attention on the use of healthy, but unwanted animals (shelter dogs, unowned stray cats, abandoned underperforming racing hounds or racing horses, etc.), their situation of being unwanted is primarily a matter of (bad) luck, which should not weigh on the decision to collect their bodies for veterinary education. If anything, this situation should on the contrary call for greater moral consideration, as these individuals are more vulnerable than their “wanted” counterparts. A useful comparison can be made here with the use of homeless people for medical education: their situation does not preclude the integration of the same ethical principles as for non-homeless people. On the contrary, because homeless people have an increased likelihood of being morally wronged, we have an increased moral duty towards them, not a decreased one [12]. Therefore, having the same rationale in mind for domestic species, it can be said that the property of being a shelter or unwanted animal does not give a moral blank check in the context of collecting their bodies and cadavers for veterinary education.

In this case, Beauchamp and Childress’ principles of biomedical ethics [13], and more especially the principles of beneficence and non-maleficence, offer a relevant framework for ethical reflection. Beneficence can be understood as a positive requirement for medical practitioners to provide some benefits to the patient and to balance these benefits with the risks and costs of the medical act in order to produce the best overall results [13]. On the other hand, non-maleficence builds on the Hippocratic maxim *primum non nocere* and obligates medical practitioners to abstain from causing harms to others [13]. As euthanasia is a medical act, the principle of beneficence requires the veterinarian performing it to justify it in terms of the best interests of the animal. Death being in the best interests of the animal is a key element that differentiates between euthanasia in the strict sense (from Ancient Greek *ευ*-, “good” and *θάνατος*, “death”) and humane killing [14–15]. Yet, *pro tanto*, such a justification would be difficult to find in the case of killing a healthy but unwanted animal. Even further, it can be argued that not only it is not in the best interests of the animal (principle of beneficence), but it also actually goes *against* the interests of the animal by harming them (principles of non-maleficence). Indeed, such animals are still likely to live a life that is worth living and this interest does not appear to be overridden by the need of obtaining the cadaver for education (precisely because the latter can be met with other options that are more justifiable from an ethical viewpoint).

The same kind of argument can be made concerning purpose-bred animals: killing these animals just because they have been purposely bred for veterinary education is not a relevant justification and is contrary to the principles of beneficence and non-maleficence. To a certain extent, the implications for purpose-bred animals are even more cynical. From a practical point of view, if we consider for the sake of the argument that animal cadavers are indeed purely “materials” for veterinary education, breeding some animals for this sole purpose can be considered as a waste of resources, as such “materials” can be gathered from the number of domestic animals that are dying at veterinary practices or at their place of residence.

Both the case of unwanted animals and the case of purpose-bred animals raise a similar ethical concern: they do not follow the basic principles of beneficence and non-maleficence that should be at the core of the education and practice of veterinarians. Following this, there are some good moral reasons to believe that either case is not a morally acceptable source of cadavers. This leads us to state a first requirement for an ethical collection of cadavers for veterinary education, the *principle of medical justification to donate*: “If an animal is alive, then there should be a medically sound reason to proceed with euthanasia (in a strict sense) before collecting the cadaver for veterinary education”. It is worth noting that a body donation program may also go against such a principle. For instance, a dog owner may want to abandon their dog and, to this end, to donate the body for veterinary education. This would be a request for “convenience euthanasia” [15–16] and would be as questionable as the case of humanely killing unwanted or purpose-bred animals because the requirements for true euthanasia are not met. But the key element to consider here is a matter of scale: it seems reasonable to think that such a case would be more the exception rather than the typical case of body donation programs. But in the case of unwanted or purpose-bred animals, the absence of consideration for the principle of medical justification to donate seems to be the general rule rather than an exception. Therefore, if neither unwanted nor purpose-bred animals are morally acceptable sources of cadavers, then only cadavers that have been donated can be used. This conclusion closely resonates with the definition of an ethically-sourced cadaver proposed by Knight [17], where these cadavers are “obtained from animals that have been euthanized for medical or severe and intractable behavioral reasons, or that have died naturally or in accidents”.

## Being dead is not enough: a case study

The emphasis on body donation programs as being the sole acceptable source of cadavers in veterinary education has been advocated for more than two decades [17–20], yet only slow-moving efforts to completely move away from the use of shelter or purpose-bred animals in veterinary dissection and surgery courses have been made so far. Fortunately, more and more body donation programs are now implemented in veterinary faculties, e.g., in the UK (University of Surrey [21], University of Bristol [22], University of Glasgow [23]), Canada (University of Calgary [24]), Australia (University of Sydney [25], University of Queensland [26]), Thailand (Chulalongkorn University [27]), the US (Tufts University [28], Texas A&M University [29]), and the Netherlands (Utrecht University [30]). Some scarce empirical evidence shows a preference for this option among veterinary students [7–8]. However, a survey in dog owners also showed that many were unaware of this possibility for their pet and that body donation programs should therefore be better advertised to veterinary clients [31].

But what does it imply exactly to *donate* a cadaver for veterinary education? We have seen that, for a living animal, there should be a medically sound reason for euthanasia before collecting the cadaver (principle of medical justification to donate). And sometimes a domestic animal may also just die from a natural cause or due to an accident. These are ethical sources of cadavers [17] and collecting cadavers for veterinary education in these contexts does not seem, at first glance, to pose additional ethical problems. But we would like to argue otherwise. To illustrate our point, here is a fictional case study:

A nine-year-old female Bernese Mountain dog is presented to an emergency service with abdominal pain and nonproductive vomiting. Abdominal radiographs are performed, showing a gastric dilatation-volvulus syndrome. Gastric dilatation-volvulus is a life-threatening condition where the stomach twists acutely on its mesenteric axis [32]. It requires emergency surgery and carries a guarded prognosis; mortality rates being estimated around 50% overall and around 10–23% after surgery [32]. Due to the advanced age of the dog, its poor health condition, the necessity of an expensive surgery and the poor prognosis associated with this medical condition, the owners choose to euthanize their dog and ask for the incineration of the cadaver. The euthanasia is eventually performed but, after the owners’ departure, the veterinarian chooses to put the cadaver on the surgery table in order to gain experience in the specific surgical procedure of gastric dilatation-volvulus.

We can first mention that this case study would comply with the principle of medical justification to donate. But two additional ethical issues can be identified in this situation. First, there is a breach of professional integrity from the veterinarian: they did not seek nor gain consent from the owners regarding the possible use of their dog as a learning experience. More importantly, they violated their final wish concerning the fate of their dog, that is, they performed a simulated surgery on the corpse whereas the choice to incinerate the cadaver was clearly stated and approved by both parties. If reported, such a breach of professional integrity would normally lead to professional sanctions by competent regulatory bodies (e.g., the Royal College of Veterinary Surgeons in the UK). Second, a lack of respect for the dog patient has been displayed. Indeed, the fact that a cadaver no longer has a prudential value (i.e. there is no self-interest for well-being) does not mean that it lacks any value *at all* and that any kind of action becomes morally permissible. As explained by Winkelmann [33], the veterinarian in this case showed a “pragmatic materialism” concerning the dog’s body and medical condition, where the cadaver is considered as mere material such as a block of wood or a technical device. For proponents of deontological arguments like Winkelmann, pragmatic materialism does not justify erasing all ethical considerations towards the late individual, and the value of a dead body cannot be completely reduced to that of an object. A cadaver, even if it has no prudential value, still calls for a certain kind of responsibility. Another approach to this question lies within the ethics of care and the relational value that a cadaver has with other moral patients. Indeed, in the ethics of care, the caring relationship extends beyond the clinical setting to also include the grieving family and the empathetic feelings of veterinarians [34]. In that sense, the virtue of care in the context of body donation creates a special responsibility for the veterinarian to treat the body with respect and sensitivity in an effort to acknowledge the bonds and relationships that have been lost. Accordingly, compliance with the principle of medical justification to donate is not enough to ethically collect a cadaver for veterinary education: there should be more considerations to it, including at least the consent from the owner and a sense of respect for the donated cadaver. In order to inform this discussion, we can turn our attention to the case of human body donation programs.

## Requirements to donate a human cadaver

In human medicine, standard practice exists within body donation programs. The use of unclaimed cadavers from unknown provenance, criminals, homeless people or grave robbing no longer occurs in most countries [35]. A 2020 review outlined the ethical recommendations of body donation in the context of human dissection [11]. It emphasizes body donation programs as the unique acceptable source of human cadavers, as stated by the International Federation of Association of Anatomists [9–10]. Three core ethical values transpire from these recommendations: informed consent from the donor or from their legal next-of-kin, absence of any commercial tie, and respect for all people involved in the donation contract (including family members). More specific considerations may also be included (see, e.g., [10–11]) but are not critical for the argumentation of this article.

Informed consent is the practical expression of a patient’s autonomy, respect for which is one of the core principles of biomedical ethics [13]. The 1914 court decision of Cardozo on the topic of medical autonomy is particularly resonating in the context of body donation: “Every human being of adult years and sound mind has a right to determine what shall be done with his own body” [36]. Hence, informed consent is made to protect the right for the patient to exercise their medical autonomy. However, and it is a matter of importance for this article, informed consent is legitimate if, and only if, the patient is competent to make informed decisions. This means that, for competent people, the decision to donate (or the decision to refuse to donate) one’s body should be made a priori during the lifetime. In some countries (e.g. Switzerland, Australia or the US), in cases of a lack of competence or death, the legal next-of-kin may make the decision on behalf of the individual.

The principle of absence of any commercial tie yields more clarification, because it actually only concerns body materials. Indeed, what is considered as unethical practice is the commercialization of human remains in the context of body donation. The reason for this, which is discussed not only for body donation but also for organ donation, is based on a Kantian argument that commercializing cadavers or body parts is a breach in the respect for the inherent value of the individual [37–38]. Therefore, not *all kinds* of commercialization in the context of body donation are problematic, only the possibility to profit from the altruistic and willing choice to donate a body. This means that, as long as no profit is made out of the body remains, costs for maintaining the body donation program, or costs for preparing, transporting, and preserving cadavers may be appropriate [9].

The third ethical principle of respect for all people involved in the donation contract concerns how transparent the future of the donated cadaver should be. The donor’s family ought to be informed about (and consent for) the way the cadaver will be collected, what types of procedures will be carried out on it, and how the remains will be disposed. Transparency can also “include providing criteria for rejecting bodies, what bodies will be used for, and the length of time body parts will be retained” [10].

## What are the requirements for an ethical body donation program in domestic animals?

The direct application of the principle of absence of commercial tie and the principle of respect for all people involved in the donation contract are not highly problematic in the context of veterinary practice. However, the principle of informed consent from the donor should be thoroughly adapted. Indeed, unlike the human patient, veterinary informed consent does not protect the autonomy of the animal “because of the lack of capacity of animal patients to make their own medical decisions” [39]. And, as discussed above, competence is a prime concern for informed consent. A practical solution is to consider the owners of domestic animals as suitable next-of-kins to make the decision of donating the body, despite some disanalogies with human body donation consent processes. In clinical practice, the implication of this consideration is to include the owners’ consent in every decision concerning the donated body. This consent should be informed, i.e. the owners need to have a full understanding of their course of action and of possible alternatives.

With this in mind, we are now able to suggest ethical requirements for a body donation program in domestic animals:

1. *Principle of medical justification to donate*: if an animal is alive, then there should be a medically sound reason to proceed with euthanasia (in a strict sense) before collecting the cadaver for veterinary education (this excludes “convenience euthanasia”).
2. *Principle of informed consent from the owner*: the owner should be informed, fully understand, and agree with the way their animal’s cadaver will be collected, what types of procedures will be carried out on it, and how the remains will be disposed of.
3. *Principle of absence of commercial tie*: no profit should be made from the remains of the donated animal cadaver, but costs for maintaining the body donation program, or costs for preparing, transporting, and preserving the cadaver may be appropriate.
4. *Principle of respect for all individuals involved in the donation program*: the donated cadaver, but also the wishes of the owners, should be treated with respect, and full transparency between all involved parties should be ensured.

After donation, any procedure conducted on the donated cadaver should be in accordance with what the owners consented to. The same rule should be applied concerning the disposal of remains. As we can see, transparency between the owners and the actual use of the donated cadaver is paramount. Respect is not only due to the donated cadaver, but also to the choices made by its family (in a broad sense, so it includes domestic animals as “family members”). As stressed by some authors, the family acts as advocates for the dead animal and for the special bond that remains between them [33]. Acknowledging this social feature builds trust and shows high standards of professional integrity that every veterinary practitioner should aim for. And because it is primarily the duty of veterinary faculties and specialized organizations using animal cadavers to educate veterinary practitioners, they should commit to such general principles outlined in this article. In this regard, the International Network for Humane Education (InterNICHE) is an excellent example, as it included since 2011 such principles in its policy on the use of animals and alternatives in education and training [20]. However, more efforts are still needed in institutions and in local regulations to further consider the case of cadavers for veterinary education.

## Conclusion

In this article, we started from the assumption that using animal cadavers for veterinary education is not morally wrong per se. In this context, the critical ethical points that we should consider are the provenance of such cadavers and how they are actually used. We argued for some ethical requirements for cadaver collection, especially that searching for an informed consent through a body donation program is the most acceptable way to obtain cadavers. In addition, transparency and respect for the animal cadaver in each step of the donation procedure allow an effective and ethical use of the cadaver. The absence of specific laws concerning the donation of cadavers in domestic species calls for the implementation of a legislative framework built upon these ethical requirements in order to regulate the collection of animal cadavers for educational purposes.

## Data Availability

Data sharing is not applicable to this article as no datasets were generated or analyzed during the current study.
